# The oxytocin-prostaglandins pathways in the horse (*Equus caballus*) placenta during pregnancy, physiological parturition, and parturition with fetal membrane retention

**DOI:** 10.1038/s41598-020-59085-1

**Published:** 2020-02-07

**Authors:** Anna Rapacz-Leonard, Mark Leonard, Małgorzata Chmielewska-Krzesińska, Marta Siemieniuch, Tomasz E. Janowski

**Affiliations:** 1 Department of Animal Reproduction with Clinic, Faculty of Veterinary Medicine, University of Warmia and Mazury in Olsztyn, Olsztyn, Poland; 2 University of Warmia and Mazury in Olsztyn, Olsztyn, Poland; 3 Department of Pathophysiology, Forensic Veterinary and Administration, Faculty of Veterinary Medicine, University of Warmia and Mazury in Olsztyn, Olsztyn, Poland; 4 Research Station in Popielno/Department of Immunology and Pathology of Reproduction, Polish Academy of Science, Olsztyn, Poland

**Keywords:** Zoology, Reverse transcription polymerase chain reaction, Immunoblotting, Immunohistochemistry, ELISA

## Abstract

Despite their importance in mammalian reproduction, substances in the oxytocin-prostaglandins pathways have not been investigated in the horse placenta during most of pregnancy and parturition. Therefore, we quantified placental content of oxytocin (OXT), oxytocin receptor (OXTR), and prostaglandin E2 and F2 alpha during days 90–240 of pregnancy (PREG), physiological parturition (PHYS), and parturition with fetal membrane retention (FMR) in heavy draft horses (PREG = 13, PHYS = 11, FMR = 10). We also quantified OXTR and prostaglandin endoperoxide synthase-2 (PTGS2) mRNA expression and determined the immunolocalization of OXT, OXTR, and PTGS2. For relative quantification of OXT and OXTR, we used western blotting with densitometry. To quantify the prostaglandins, we used enzyme immunoassays. For relative quantification of OXTR and PTGS2, we used RT-qPCR. For immunolocalization of OXT, OXTR, and PTGS2, we used immunohistochemistry. We found that OXT was present in cells of the allantochorion and endometrium in all groups. PTGS2 expression in the allantochorion was 14.7-fold lower in FMR than in PHYS (*p* = 0.007). These results suggest that OXT is synthesized in the horse placenta. As PTGS2 synthesis is induced by inflammation, they also suggest that FMR in heavy draft horses may be associated with dysregulation of inflammatory processes.

## Introduction

In the placenta, a delicate balance of hormones helps to support pregnancy and initiate parturition^1–3^. Parturition is a proinflammatory event^4–8^, and delivery of the fetus and the placenta are accompanied by coordinated contractions, pain, and swelling, which are mostly mediated by prostaglandins^9^.

Exposure of the placenta to endogenous or exogenous oxytocin (OXT) causes it to release prostaglandin E2 (PGE_2_) and prostaglandin F2 alpha (PGF_2ɑ_) within a few hours^10–21^ (see Supplementary Fig S1 for the pathways that start with OXT release and end with the prostaglandins binding to their receptors). OXT is released from the posterior pituitary gland^22^, and during pregnancy and parturition in humans, from the placenta^23^. Given that, of all domestic and laboratory species, horses may be the most similar to humans in terms of the endocrinology of pregnancy and parturition^24^, it seems likely that horses also release OXT from the placenta. Moreover, human placental OXT is not believed to play a role in myometrial contractions, unlike pituitary OXT^13^. Thus, it seems likely that horse placental OXT would be a signal for prostaglandin release in the placenta itself, and not a signal for myometrial contractions. However, the presence of OXT in horse placental cells needs to be verified.

OXT exerts its effects by binding with its receptor (OXTR), which is expressed in the myometrium, mammary gland, and placenta^23^. During pregnancy and parturition in horses, OXTR has only been studied in the nonpregnant uterus, the endometrium in early pregnancy (<21 days)^16,25,26^, and the endometrium and allantochorion within 3 hours of foal delivery^27^. Thus, OXTR levels in the horse placenta during the majority of pregnancy and parturition remain unknown.

The chemical precursor of prostaglandins, arachidonic acid, is abundant in cell membranes. This means that the rate limiting step in production of PGE_2_ and PGF_2α_ is the conversion of arachidonic acid to prostaglandin H2 by prostaglandin endoperoxide synthase-1 and prostaglandin endoperoxide synthase-2 (PTGS1 and PTGS2, formerly referred to as cyclooxygenase-1 and 2). PTGS2 is the predominant isoform in human, bovine, and ovine placentas^18,19,28^. PTGS2 synthesis is induced by inflammation^29–34^, and during pregnancy in the horse, its synthesis should be suppressed by high levels of progestagens, as it is suppressed by high levels of progesterone in other species^35^. Similarly, it seems likely that PTGS2 synthesis would be upregulated at parturition in the horse, as it is in other species. However, neither PTGS2 enzyme nor PTGS2 mRNA have been quantified in the horse placenta after day 22 of pregnancy or at parturition.

During pregnancy and parturition, PGE_2_ and PGF_2α_ are synthesized by the placenta^35,36^ and immediately secreted^28^. In epitheliochorial placentas, like that of the mare, they are believed to be synthesized mostly in the allantochorion and quickly degraded in the endometrium^35^. Both prostaglandins are released to placental fluids, but only small quantities are released to maternal peripheral blood^35^. Thus, for better insight into pregnancy and parturition in horses, it would be helpful to directly measure the content of PGE_2_ and PGF_2α_ in the placenta.

The current thinking on PGE_2_ and PGF_2α_ in reproduction emphasizes not only their uterotonic roles, but also their proinflammatory effects. In addition to being a uterotonic^7^, PGE_2_ causes cervical ripening in humans^37^, and it has been effective as a drug for opening the cervix in horses^38^. It increases pain, swelling, and temperature. It also increases the migration of immune cells toward placental tissues, and these cells play important roles in tissue remodelling, including activation of matrix metalloproteinases^4,7,37,39,40^. PGF_2α_ is not only the main uterotonic agent in many species^23,35^, but it also augments the inflammatory cascade in the placenta by increasing output of interleukins and chemokines^40^.

Dysregulation of tissue remodelling and inflammatory processes may play roles in fetal membrane retention (FMR) in horses, as indicated by our previous findings that extensive placental fibrosis^41^, and differential placental content and activity of matrix metalloproteinases^42^ are associated with FMR in heavy draft horses. This led us to hypothesize that, in the placenta at foal delivery, FMR in this breed is associated with differential content of substances in the OXT-prostaglandins pathways and differential expression of genes coding for these substances. Given that FMR is the most common peripartum condition in horses, particularly in heavy draft horses^41,43^, and that FMR can be life-threatening in this species, it seemed worthwhile to test this hypothesis.

Therefore, we had two primary objectives: first, to quantify the content of four key substances in these pathways (OXT, OXTR, PGE_2_, and PGF_2α_) in the allantochorion and the endometrium of heavy draft horses during days 90–240 of pregnancy, physiological parturition, and FMR. Second, to provide further information about these pathways by quantifying mRNA expression of OXTR and PTGS2 in these tissues in these groups. For further context, we also determined the immunolocalization of OXT, OXTR, and PTGS2. To quantify mRNA, proteins/peptides, and prostaglandins, we used RT-qPCR, western blotting with densitometry, and enzyme immunoassays, respectively. For immunolocalization of OXT, OXTR, and PTGS2, we used immunohistochemistry.

## Results

### OXT

**Figure 1:**
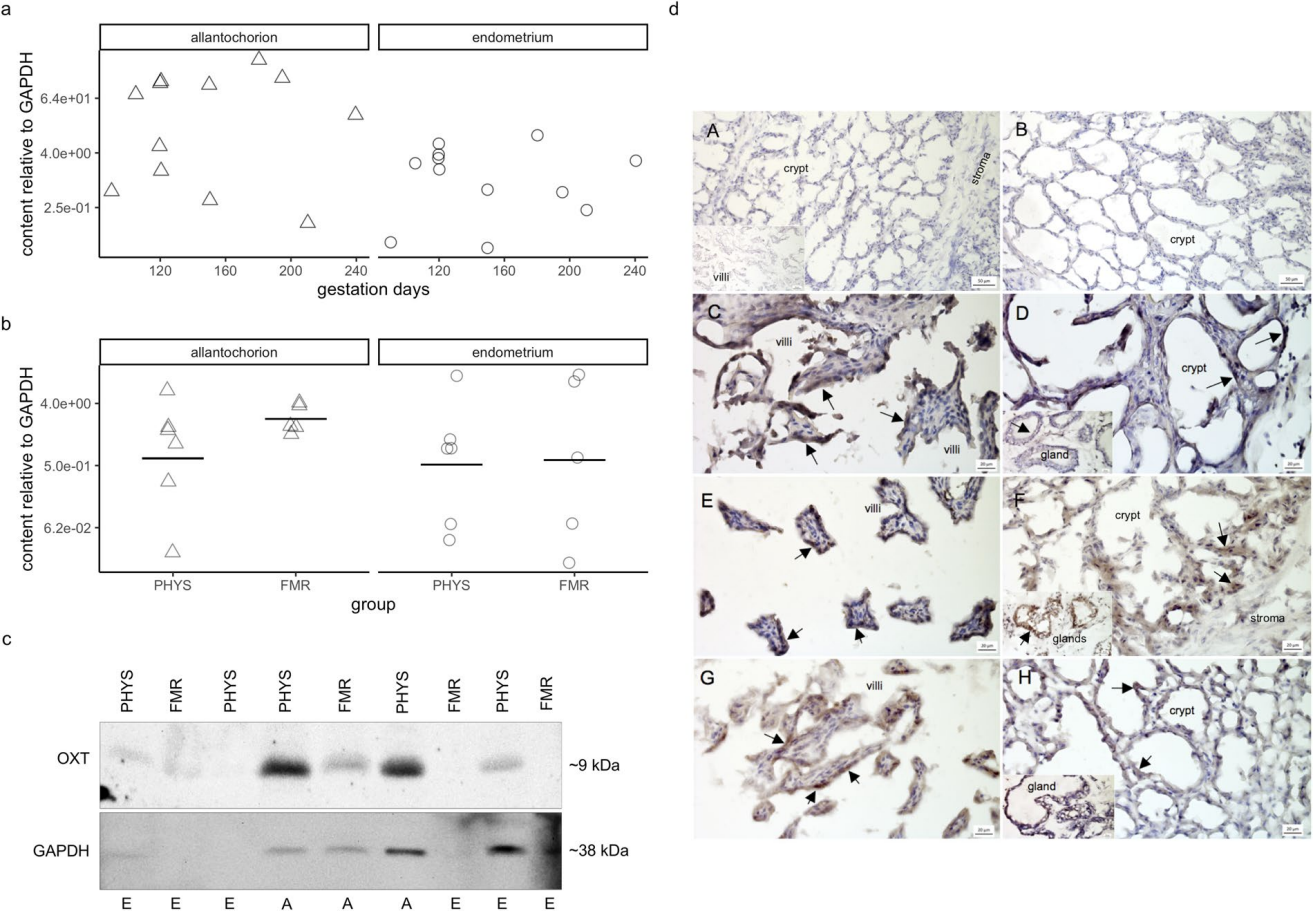
Oxytocin peptide content and immunolocalization. (**a**) Oxytocin peptide content relative to that of glyceraldehyde-3-phosphate dehydrogenase (GAPDH) during pregnancy. Protein content was quantified by western blotting with densitometry. Symbols correspond to tissues from individual mares (Δ, allantochorion; ○, endometrium). Note the logarithmic scales in panels a and b. (**b**) Oxytocin peptide content relative to that of GAPDH in physiological parturition (PHYS) and parturition with fetal membrane retention (FMR). Horizontal lines indicate geometric means. (**c**) Representative blots of oxytocin and GAPDH. Uncropped blots are presented in Supplementary Fig S4. Image Lab version 5.2.1 software was used for visualization and densitometry (Bio-Rad Laboratories, https://www.bio-rad.com/en-pl/product/image-lab-software?ID=KRE6P5E8Z). Abbreviations: OXT, oxytocin; A, allantochorion; E, endometrium. (**d**) Tissue expression and localization of oxytocin in horse placenta. Oxytocin peptide was visible as dark brown to black cytoplasmic staining (arrows). Images of negative controls (**A**,**B**): (**A**) endometrium with primary antibody omitted (no staining visible); inset in (**A**), allantochorion with primary antibody omitted (no staining visible); (**B**) endometrium stained with antibody incubated with blocking peptide (no staining visible). Images of the pregnancy group (**C**,**D**): (**C**) allantochorion with positively stained epithelial cells on villi; (**D**) endometrium with positively stained epithelial cells in crypts; inset in (**D**), endometrial glands with positively stained endothelial cells. Images of the physiological parturition group (**E**,**F**): (**E**) allantochorion with positively stained epithelial cells on villi; (**F**) endometrium with positively stained epithelial cells in crypts; inset in (**F**), endometrial glands with positively stained endothelial cells. Images of the fetal membrane retention group (**G**,**H**): (**G**) allantochorion with positively stained epithelial cells on villi; (**H**) endometrium with positively stained epithelial cells in crypts; inset in (**H**), endometrial glands with positively stained endothelial cells. Micrographs were made with Zen 2012 (blue edition) software (Zeiss, https://www.zeiss.com/microscopy/int/products/microscope-software/zen-lite.html).

In pregnancy, there was no apparent correlation between OXT peptide content and gestation days (allantochorion: τ = 0.08, *p* = 0.89; endometrium: τ = −0.05, *p* = 0.92) (Fig. 1a).

Mean OXT content was higher in FMR than in physiological parturition in both tissues, but the differences were not statistically significant (allantochorion: 3.7-fold, 95% confidence interval for the fold-change (CI) = −1.9 to 27.0-fold, *p* = 0.44; endometrium: 1.2-fold, CI = −30.1 to 41.1-fold, *p* = 0.92) (Fig. 1b). The variation in OXT content within groups was large relative to the difference between group means, with the exception of the allantochorion in FMR, in which there was relatively little difference between horses.

Interestingly, immunohistochemical staining for OXT was visible in the cytoplasm in both tissues in all groups (Fig. 1d). It was present mostly in the epithelial cells of villi and endometrial crypts. In the endometrium, OXT was also visible in the endothelial cells of the glands.

### OXTR

**Figure 2:**
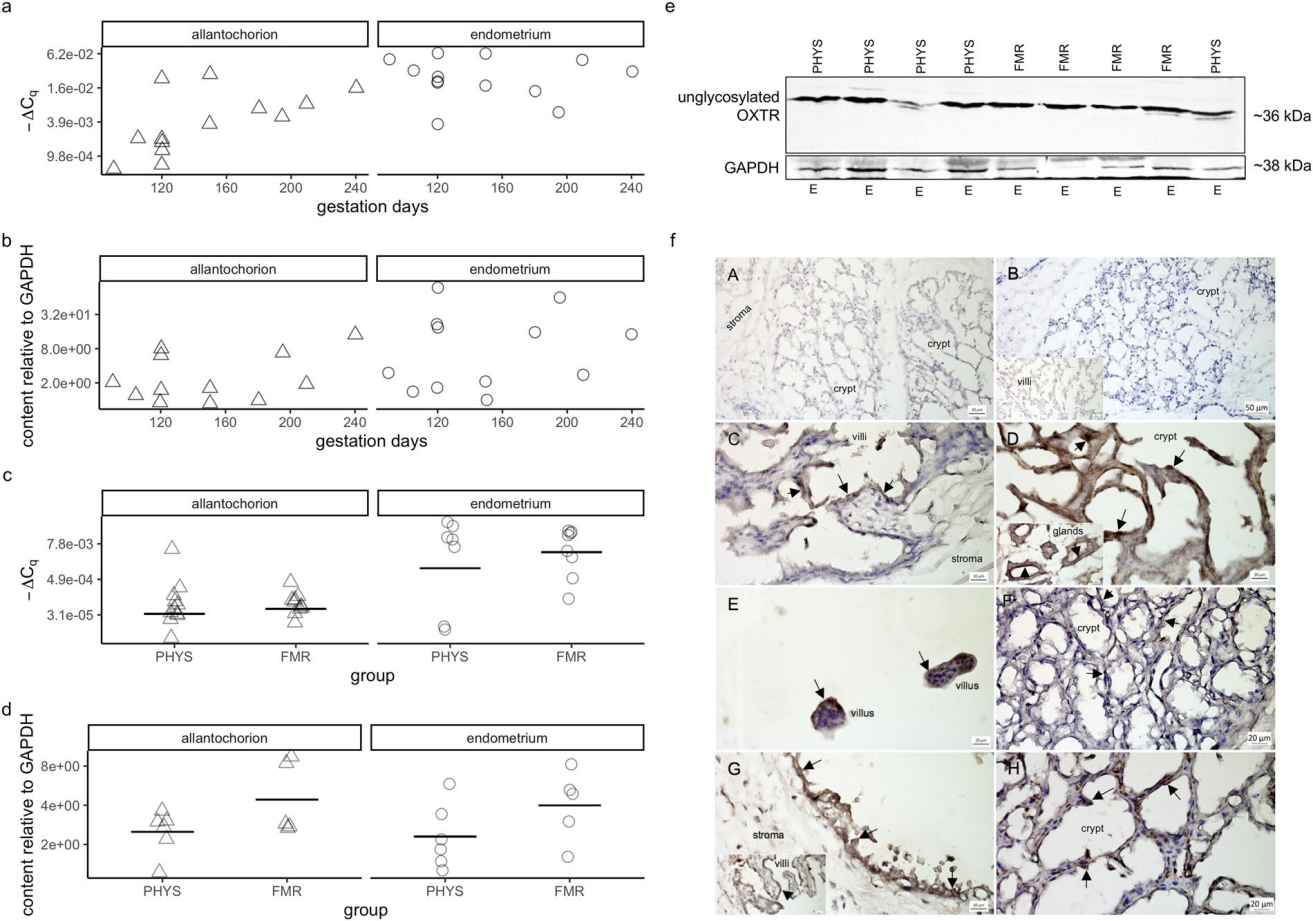
Oxytocin receptor (OXTR) mRNA expression, protein content, and immunolocalization. (**a**) OXTR mRNA expression during pregnancy. mRNA expression was quantified by RT-qPCR. ∆C_q_ is the difference between the quantification cycle value of OXTR and that of glyceraldehyde-3-phosphate dehydrogenase (GAPDH) after weighting those values by the genes’ amplification efficiencies. Symbols correspond to tissues from individual mares (Δ, allantochorion; ○, endometrium). Note the logarithmic scales in panels a–d. (**b**) OXTR protein content relative to that of GAPDH during pregnancy. Protein content was quantified by western blotting with densitometry. (**c**) OXTR mRNA expression in physiological parturition (PHYS) and parturition with fetal membrane retention (FMR). Horizontal lines indicate geometric means. (**d**) OXTR protein content relative to that of GAPDH in PHYS and FMR. (**e**) Representative blots of OXTR and GAPDH. Uncropped blots are presented in Supplementary Fig S5. Quantity One 1-D version 4.6.6 software was used for visualization and densitometry (Bio-Rad Laboratories, https://www.bio-rad.com/en-pl/product/quantity-one-1-d-analysis-software?ID=1de9eb3a-1eb5-4edb-82d2-68b91bf360fb). Abbreviation: E, endometrium. (**f**) Tissue expression and localization of OXTR in horse placenta. Oxytocin receptor protein was visible as dark brown to black membrane staining (arrows). Hematoxylin was used as a counterstain. Images of positive and negative controls (**A**,**B**): (**A**) endometrium with primary antibody omitted (no staining visible); (**B**) endometrium stained with antibody incubated with blocking peptide (no staining visible); inset in (**B**), allantochorion stained with antibody incubated with blocking peptide (no staining visible). Images of the pregnancy group (**C**,**D**): (**C**) allantochorion with positively stained epithelial cells on villi; (**D**) endometrium with positively stained epithelial cells in crypts; inset in (**D**), endometrial glands with positively stained endothelial cells. Images of the physiological parturition group (**E**,**F**): (**E**) allantochorion with positively stained epithelial cells on villi; (**F**) endometrium with positively stained epithelial cells in crypts. Images of the fetal membrane retention group (**G**,**H**): (**G**) allantochorion with positively stained epithelial cells; inset in (**G**), allantochorion with positively stained epithelial cells on villi; (**H**) endometrium with positively stained epithelial cells in crypts. Micrographs were made with Zen 2012 (blue edition) software (Zeiss, https://www.zeiss.com/microscopy/int/products/microscope-software/zen-lite.html).

In the allantochorion in pregnancy, OXTR mRNA expression tended to increase with gestation days (Fig. 2a). This correlation was moderately strong but not statistically significant (τ = 0.54, *p* = 0.13). However, there was little correlation between gestation days and OXTR expression in the endometrium, or between gestation days and OXTR protein content in the allantochorion and endometrium (τ = −0.18, *p* = 0.68; τ = 0.18, *p* = 0.68; τ = 0.08, *p* = 0.89; respectively) (Fig. 2a,b).

In both tissues, mean OXTR expression was higher in FMR than in physiological parturition, but the differences were not significant (allantochorion: 1.5-fold, CI = −1.4 to 3.2-fold, *p* = 0.59; endometrium: 3.5-fold, CI = −8.3 to 102.1, *p* = 0.68) (Fig. 2c). Similarly, in both tissues, OXTR content was higher in FMR, although the differences were not significant (allantochorion: 1.8-fold, CI = −1.2 to 3.9-fold, *p* = 0.89; endometrium: 1.7-fold, CI = −1.3 to 4.0-fold, *p* = 0.44) (Fig. 2d). The variation in OXTR expression and OXTR content within groups was large relative to the difference between group means.

Immunohistochemical staining for OXTR was visible on the cell membrane (Fig. 2f). In the allantochorion, it was present on the epithelial cells of the villi, and also on the endothelial cells of some vessels. In the endometrium, it was present on the epithelial cells of crypts and on the endothelial cells of glands and some vessels.

### PTGS2

**Figure 3:**
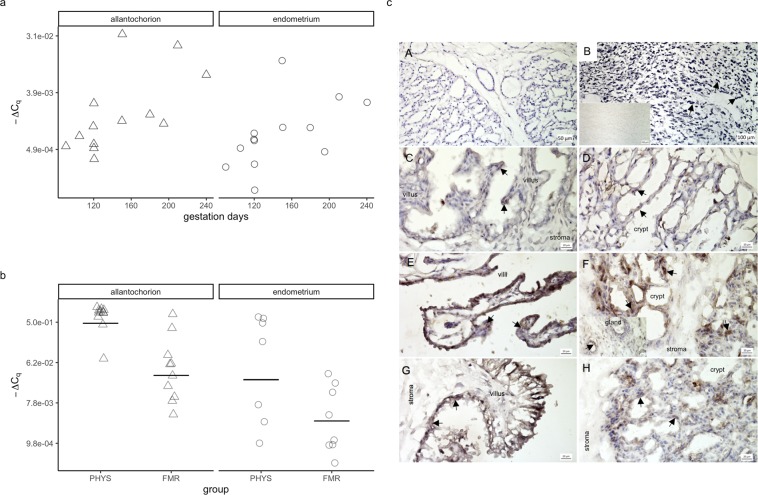
Prostaglandin endoperoxide synthase-2 (PTGS2) mRNA expression and immunolocalization. (**a**) PTGS2 mRNA expression during pregnancy. mRNA expression was quantified by RT-qPCR. ∆C_q_ is the difference between the quantification cycle value of PTGS2 and that of glyceraldehyde-3-phosphate dehydrogenase after weighting those values by the genes’ amplification efficiencies. Symbols correspond to tissues from individual mares (Δ, allantochorion; ○, endometrium). Note the logarithmic scales in panels a and b. (**b**) PTGS2 mRNA expression in physiological parturition (PHYS) and parturition with fetal membrane retention (FMR). Horizontal lines indicate geometric means. (**c**) Tissue expression and localization of PTGS2 in horse placenta. PTGS2 protein was visible as dark brown to black cytoplasmic or nuclear staining (arrows). Hematoxylin was used as a counterstain. Images of positive and negative controls (**A**,**B**): (**A**) endometrium stained with antibody incubated with blocking peptide (no staining visible); (**B**) mature corpus luteum with positively stained luteal cells; inset in (**B**), the same corpus luteum stained with primary antibody omitted (no staining visible). Images of the pregnancy group (**C**,**D**): (**C**) allantochorion with some positively stained epithelial cells on villi; (**D**) endometrium with some positively stained epithelial cells in crypts. Images of the physiological parturition group (**E**,**F**): (**E**) allantochorion with positively stained epithelial cells on villi; (**F**) endometrium with positively stained epithelial cells in crypts; inset in (**F**), endometrial glands with some positively stained endothelial cells. Images of the fetal membrane retention group (**G**,**H**): (**G**) allantochorion with positively stained epithelial cells; (**H**) endometrium with positively stained epithelial cells in crypts. Micrographs were made with Zen 2012 (blue edition) software (Zeiss, https://www.zeiss.com/microscopy/int/products/microscope-software/zen-lite.html).

In both tissues in pregnancy, PTGS2 expression tended to increase with greater gestation length (Fig. 3a). Although these correlations were not statistically significant, they were moderately strong (allantochorion: τ = 0.48, *p* = 0.19; endometrium: τ = 0.54, *p* = 0.13).

In the allantochorion, mean PTGS2 expression was substantially lower in FMR than in physiological parturition, and the difference was statistically significant (–14.7-fold, CI = −49.8 to –4.3-fold, *p* = 0.007) (Fig. 3b). In the endometrium, although the difference was not significant, PTGS2 expression was also substantially lower in FMR than in physiological parturition (–8.4-fold, CI = −105.7 to 1.5-fold, *p* = 0.32).

In both physiological parturition and FMR, staining for PTGS2 protein was mostly visible in the cytoplasm in the form of granules, and some nuclei were also stained (Fig. 3c). Some cytoplasmic staining was also visible in pregnancy. In all groups, staining was observed in epithelial cells in these locations: the endometrial crypts, the surface of the allantochorion, and the villi. Additionally, it was visible in the endothelium of endometrial glands.

### PGE_2_

**Figure 4:**
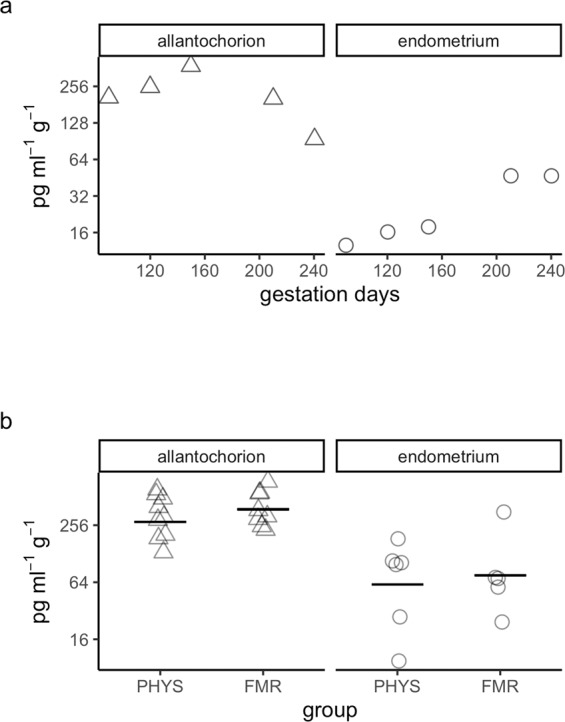
Prostaglandin E2 content. (**a**) Prostaglandin E2 content during pregnancy. Hormone content was quantified by enzyme immunoassay. Symbols correspond to tissues from individual mares (Δ, allantochorion; ○, endometrium). Note the logarithmic scales in panels a and b. (**b**) Prostaglandin E2 content in physiological parturition (PHYS) and parturition with fetal membrane retention (FMR). Horizontal lines indicate geometric means.

In the allantochorion in pregnancy, PGE_2_ content tended to decrease with gestation days. This correlation was moderately strong but not statistically significant (τ = −0.40, *p* = 0.68) (Fig. 4a). In the endometrium, PGE_2_ content showed a strong but non-significant tendency to increase with gestation days (τ = 0.80, *p* = 0.32).

In the allantochorion in FMR, mean PGE_2_ content was 1.4-fold higher than in this tissue in physiological parturition (Fig. 4b, Supplementary Table S2). Although this difference was not significant, the CI included values up to 2.4-fold higher in FMR (CI = −1.3 to 2.4-fold, *p* = 0.57). In the endometrium, PGE_2_ content was 1.2-fold higher in FMR. This difference was not significant, but the CI included values up to 5.1-fold higher in FMR (CI = −3.3 to 5.1-fold, *p* = 0.89).

### PGF_2α_

**Figure 5:**
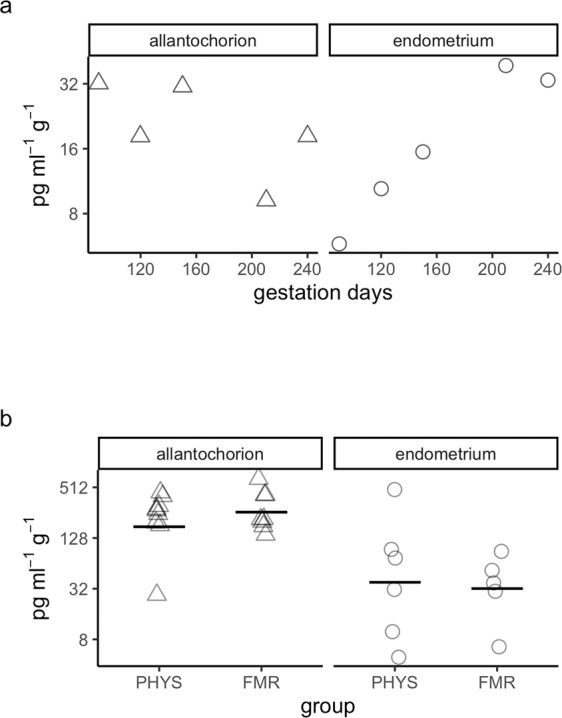
Prostaglandin F2 alpha content. (**a**) Prostaglandin F2 alpha content during pregnancy. Hormone content was quantified by enzyme immunoassay. Symbols correspond to tissues from individual mares (Δ, allantochorion; ○, endometrium). Note the logarithmic scales in panels **a** and **b**. (**b**) Prostaglandin F2 alpha content in physiological parturition (PHYS) and parturition with fetal membrane retention (FMR). Horizontal lines indicate geometric means.

In the allantochorion in pregnancy, there was a moderately strong but non-significant tendency for PGF_2α_ content to decrease with greater gestation length (τ = −0.40, *p* = 0.68) (Fig. 5a, Supplementary Table S2). In the endometrium, there was a strong but non-significant tendency for its content to increase with gestation length (τ = 0.80, *p* = 0.32).

In the allantochorion, mean PGF_2α_ content was 1.5-fold higher in FMR than in physiological parturition (Fig. 5b). This difference was not significant, but the CI included values up to 3.0-fold higher in FMR (CI = −1.4 to 3.0-fold, *p* = 0.57). In the endometrium, its content was 1.2-fold lower in FMR than in physiological parturition. Here too, the difference was not significant, although the CI included values as low as 7.3-fold lower in FMR (CI = −7.3 to 5.2-fold, *p* = 0.91).

## Discussion

Our results indicate that OXT is present within the cells of the horse allantochorion and endometrium during pregnancy, physiological parturition, and FMR. They also indicate that PTGS2 expression in the allantochorion is markedly lower in FMR than in physiological parturition.

When considering the results of our study, there are three general limitations that should be taken into account. First, we studied heavy draft horses and not other breeds, and all of our horses in physiological parturition and FMR lived in the vicinity of our practice. Thus, some of our findings may not apply to other horse populations. Nevertheless, our study is a step towards better understanding of the endocrinology of equine reproduction in general and of FMR, which is particularly common in heavy draft horses.

Second, many of our estimates of differences between pregnancy, physiological parturition, and FMR have wide CIs. This is due in part to the small number of horses in our study. This small number also meant that we could not estimate the influence of herd- and farm-level confounders on our results. However, many studies of these large and expensive-to-maintain animals have similar numbers, and combining such studies in meta-analyses should provide more precise estimates.

Third, in the parturition groups, we took samples immediately after foal delivery to avoid unnecessary risks to our patients. Therefore, our results are representative of the placental levels of substances at that time, but they do not necessarily reflect levels of those substances at earlier or later stages of parturition. Although differential protein/hormone content or gene expression that is associated with FMR at foal delivery may not be a cause of the condition, these associations can provide information about the aetiology of FMR, as we discuss below in the context of the PTGS2 results.

The presence of OXT in the cytoplasm of cells in the allantochorion and endometrium of all three groups strongly suggests that OXT is synthesized in the horse placenta. Proof of this hypothesis awaits a confirmed horse *OXT* sequence so that *OXT* expression in the placenta can be verified. However, the fact that humans, whose reproductive endocrinology is very similar to that of horses^24^, synthesize OXT in the placenta^13,23^ suggests that OXT is also synthesized in the horse placenta.

The presence of additional OXTR bands in some of our western blots, and the difference between the molecular weight of the un-glycosylated OXTR band in our study (~36 kDa) and the weights reported by other studies (~70 kDa^26^, ~55 kDa^27^) is likely due to the accuracy of molecular weight estimation with SDS-PAGE (±5–10%^44^) and differences in the glycosylation status of OXTR. Glycosylation of OXTR is common, and as a result, multiple bands are often detected^45^. The protein has three glycosylation sites^46^, and each additional glycosylation core adds ~10 kDa^45^. So far, no effect of glycosylation on OXTR function has been found^47,48^; thus, differences in its glycosylation status seem unlikely to be physiologically relevant.

Although OXTR expression and OXTR content in both tissues were higher in FMR than in physiological parturition, the differences were not statistically significant. More importantly, the within-group variation in OXTR expression, and OXT and OXTR content was large relative to the difference between group means. This suggests that most of the variation in the levels of these substances is due to factors other than the presence or absence of FMR.

Our present results differ from those of our previous study of the placenta in horses, in which we observed substantially less OXTR content in FMR associated with secondary atony of the uterus than in physiological parturition^27^. The difference between studies is attributable to two factors: first, the FMR horses in our present study did not have secondary atony of the uterus. Second, the horses in our present study were sampled immediately after foal delivery, not up to three hours after, as they were in our previous study.

After adjustment for multiple comparisons, the correlations between gestation days and PTGS2 expression in the allantochorion and endometrium were no longer significant at *p* < 0.05. However, this does not mean our data indicate that, in the population of all such horses, there is no correlation. Rather, these results should be considered inconclusive^49,50^. Some evidence from sheep suggests that PTGS2 expression in horses would tend to increase as gestation progresses, as Wimsatt^18^ found that PTGS2 activity increased close to term in maternal parts of the sheep placenta.

Our results indicate that PTGS2 expression in the allantochorion at foal delivery is markedly lower in FMR than in physiological parturition. All of the values in the CI for the difference (49.8 to 4.3-fold lower in FMR) are large enough that this difference seems likely to be biologically relevant.

PTGS2 production is induced by inflammation, and the enzyme is generally only found in inflamed tissues^29–34^. Thus, lower PTGS2 expression in FMR suggests that the condition is associated with dysregulation of inflammatory processes. The inflammatory response plays a key role in both physiological tissue repair/remodelling and in fibrosis^51,52^, and growing evidence indicates its importance in parturition^6,53^. Tissue remodelling may have particular relevance to FMR in horses. It has been hypothesized that, in this species, shrinkage of the allantochorion villi allows them to slide out of their corresponding endometrial crypts, thus releasing the fetal membranes^54^. This shrinkage would involve tissue remodelling. Previously, we found that extensive fibrosis^41^, and differential content and activity of matrix metalloproteinases^42^ are associated with FMR in heavy draft horses. Thus, evidence from this study and others suggests that, in this breed of horse, FMR is linked to dysregulation of inflammation and tissue remodelling.

Although the results were statistically inconclusive (*p* ≥ 0.32), the correlations between gestation days and content of PGE2 and PGF2a ranged from moderately strong to strong. Interestingly, the content of both prostaglandins in the allantochorion tended to decrease with gestation days, whereas their content in the endometrium tended to increase. The data on prostaglandin content in FMR and physiological parturition were also inconclusive, but the CIs contained differences large enough to be physiologically relevant. Moreover, because PTGS2 expression in the allantochorion was lower in FMR than in physiological parturition, the content of prostaglandins in this tissue may decrease faster in FMR than in physiological parturition as the third stage of parturition progresses. Thus, it would be worthwhile to conduct further investigations of prostaglandin content during pregnancy, physiological parturition, and FMR, and to combine the results of all such studies in a meta-analysis.

In summary, our study indicates that OXT is present in the cells of the horse placenta during pregnancy, physiological parturition, and FMR, and that PTGS2 expression in the allantochorion is lower in FMR than in physiological parturition. The presence of OXT in these cells suggests that the horse placenta synthesizes OXT. Lower PTGS2 expression in FMR suggests a link between this condition and dysregulation of inflammatory processes in heavy draft horses.

## Material and Methods

### Settings, animals, and sample collection

All animals were heavy draft horses. Material from some of the samples that were taken for this study was also used to describe the expression of major histocompatibility complex class I in the equine placenta^55^.

To standardize sampling, we took all samples from the same place in the body of the pregnant uterus, either immediately post-mortem in pregnancy or intra-vitality in physiological parturition and FMR. We took samples from this location because it was the furthest that we could reach with equine biopsy forceps. The Local Ethics Committee for Experiments on Animals in Olsztyn (Poland) approved all sample collection protocols (permission no. 05/2014/DTN for slaughterhouse samples and 18/2015/DTN for patient samples), and we followed guidance in EU Directive 2010/63/EU for animal experiments.

### Pregnancy

Samples were obtained post-mortem from 13 horses in a slaughterhouse (the pregnancy status of the mares was not known until after slaughter). See Table 1 for the number of horses assayed with each technique. The duration of pregnancy (3–8 months) was determined based on fetal development^56^, but the ages of the mares were not available to us. Three biopsies of allantochorion were taken, and separately, three biopsies of endometrium. The horses were healthy before slaughter, and placental inflammation was not detected.

**Table 1:** Number of horses assayed with each technique.

Group	PREG	PHYS	FMR
Method	allantochorion/ endometrium	allantochorion/ endometrium	allantochorion/ endometrium
RT-qPCR	13/13	11/7	10/8
EIA	5/5	9/6	8/5
Western blotting	12/12	6/6	5/5
Immunohistochemistry	6/6	6/6	6/6

Abbreviations: PREG, pregnancy; PHYS, physiological parturition; FMR, fetal membrane retention; RT-qPCR, reverse transcription quantitative polymerase chain reaction; EIA, enzyme immunoassay.

### Physiological parturition and FMR

All mares in these groups (physiological parturition, 11 mares; FMR, 10 mares; see Table 1 for the number of horses assayed with each technique) were monitored during their entire pregnancy; none of them developed placentitis. They weighed approximately 800–900 kg; physiological parturition mares had a mean age of 7.6 years (range 3–15); FMR mares had a mean age of 10.0 years (range 4–16). All mares delivered foals within the timeframe for term delivery^57^, and the second phase of parturition lasted for 10-20 minutes.

Directly after foal delivery, uterine biopsies were taken as described in detail in Rapacz-Leonard^42^ and immediately fixed (fixation details are below). Because some owners feared that taking samples from the endometrium might decrease fertility, fewer horses were biopsied in the endometrium than in the allantochorion (Table 1). The myometrium was not biopsied to avoid unnecessary risks to our patients. To control for a possible effect of sampling on placenta retention, each horse had four biopsies taken from each sampled tissue. In general, mares with a history of physiological delivery delivered physiologically, and mares with a history of FMR retained the placenta, but two mares with a history of FMR did not retain after endometrial sampling.

Physiological parturition mares delivered fetal membranes within 20–120 minutes. If fetal membranes were not expelled within 180 minutes of foal delivery, mares were classified as having FMR^43,58–60^, then manually examined and treated.

### RT-qPCR

Samples were fixed in RNA*later* Stabilization Solution (ThermoFisher Scientific) and stored at −80 °C until assay. 50 mg of tissue per sample was used. RNA was isolated with a Total RNA Mini Kit (A&A Biotechnology). RNA concentration and quality were determined using a NanoVue Plus Spectrophotometer (GE Healthcare). After DNase treatment, reverse transcription was performed with a Maxima First Strand cDNA Synthesis Kit (ThermoFisher Scientific).

Primers were designed using Primer-BLAST and manufactured by Sigma-Aldrich (Table 2). No secondary structures were detected (Supplementary Fig S2 and S3); no pseudogenes were amplified. To confirm primer specificity, PCR products were sequenced (Supplementary Table S3). As negative controls, reactions without template, DNA polymerase, or reverse transcriptase were used, as well as a reaction with only ultrapure water.

**Table 2:** Primers for RT-qPCR analysis.

Gene name and abbreviation	Sequence accession number	Primers	Sequence (5′ → 3′)	Amplicon length	Location by exon	Concentration	Efficiency
*GAPDH* as a reference gene^75,76^	NM_001163856.1	Forward Reverse	GTCAAGCTCATTTCCTGGTATGAC TTGCTGGGTGATTGGTGGTC	117	Yes, exon-exon junction by exons no. 11 and 12	F: 5μM R: 5 μM	86.4%
*OXTR*	XM_023620040.1 variant X1 XM_023620041.1 variant X2 XM_014731360.2 variant X3	Forward Reverse	TTCATCATCGTGCTGGCCTT TGAAAGCCGAGGCTTCCTTG	103	Yes, exon-exon junction by exons no. 3 and 4 (for X1), and exons no. 2 and 3 (for X2 and X3)	F: 5μM R: 5 μM	114.3%
*PTGS2*	NM_001081775.2	Forward Reverse	TGGGTCACGGGGTGGATTTA GCGGATACACCTCGCCATTA	120	Yes, exon-exon junction by exons no. 5, 6, and 7	F: 5μM R: 5 μM	115.2%

Abbreviations: GAPDH, glyceraldehyde-3-phosphate dehydrogenase; OXTR, oxytocin receptor; PTGS2, prostaglandin-endoperoxide synthase 2.

RT-qPCR was performed with SYBR Select Master Mix (ThermoFisher Scientific) using a 7500 Fast Real-Time PCR System (Applied Biosystems). The reaction volume was 20 µL (10 µL Master Mix, 8 µL ultrapure water, 1 µL cDNA (~75 ng), 1 µL primer mix). The efficiency-corrected expression of the investigated genes relative to that of GAPDH was calculated^61^.

### Western blotting

Samples were fixed in liquid nitrogen and stored at −80 °C until assay. 100 µg of protein were loaded in each well.

For quantification of OXT, anti-OXT antibody (Table 3) was used. Because OXT was present in small amounts, chemiluminescent western blotting with horseradish peroxidase was employed^44^. The procedure in Rękawiecki^62^ was followed, with three modifications: Mini-Protean TGX Stain-Free Precast Gels 4–20% (456–8093, Bio-Rad) were used, as well as semi-dry transfer with Sequi-Blot PVDF Membrane (162–0184, Bio-Rad) and Prestained SDS-PAGE Standards (161–0318, Bio-Rad). Briefly, after electrophoresis, the transfer was performed with Tris-glycine methanol buffers at 0.2 A for 45 min. After the transfer, non-specific binding was blocked with 5% nonfat dry milk in TBST buffer. The membranes were then left overnight to incubate with primary antibody.

**Table 3:** List of antibodies.

Name of the antibody	Supplier and catalogue no.	Antibody ID	Host	Clonality	Antigen	Validated by western blot	Used at concentration	Available blocking peptide	Supplier and catalogue no. for blocking peptide	Used at ratio
Anti- OXT	LifeSpan BioScience LS-C145973	AB_10969170	rabbit	polyclonal	aa11-60 of human OXT (P01178, NP_000906)	yes	1ug/1 ml TBST	yes	LifeSpan BioScience LS-E15388	4:1
Anti- OXTR	Abcam ab87312	AB_10674457	goat	polyclonal	aa 355–367 of human OXTR (C terminal) Sequence: C-RRLGETSASKKSN	yes	5ug/1 ml TBST	yes	Abcam ab173096	3:1
Anti-PTGS2	Santa Cruz Biotechnology sc-1746	AB_631310	goat	polyclonal	peptide mapping near the N-terminus of Cox −2 of rat origin	yes by^77,78^	1:100	yes	Santa Cruz Biotechnology sc-1746P	4:1
Anti-GAPDH	Sigma-Aldrich G8795	AB_1078991	mouse	monoclonal	hybridoma GAPDH-71.1 produced by the fusion of mouse NS1 cells and splenocytes from BALB/c mice immunized with rabbit GAPDH	yes	2ul/24 ml TBST	no		

Abbreviations: GAPDH, glyceraldehyde-3-phosphate dehydrogenase; OXT, oxytocin; OXTR, oxytocin receptor; PTGS2, prostaglandin-endoperoxide synthase 2.

For OXT visualization, (Sigma-Aldrich Cat# A6154, RRID:AB_258284, 0.5ul/25 ml TBST) was used. Then blots were stripped and incubated with anti-GAPDH antibody (Table 3). For GAPDH visualization, (Santa Cruz Biotechnology Cat# sc-2318, AB_641171, 0.75ul/15 ml TBST) was used.

To quantify OXTR, which was present in abundance, colorimetric western blotting with alkaline phosphatase was used^44^, following the procedure in Kowalik^63^ except for the use of 10% polyacrylamide gel electrophoresis with SDS (SDS–PAGE). Briefly, after electrophoresis with PageRuler Prestained Protein Ladder (SM0671, Fermentas), wet transfer was performed with Tris-glycine methanol buffer on ice at 60 V for 90 min. Blocking and incubation with antibody were performed as for OXT. To detect OXTR, anti-OXTR antibody (Table 3) was used with (Sigma-Aldrich Cat# A4187, RRID:AB_258141, 1ul/30 ml TBST). After visualization, blots were incubated with anti-GAPDH antibody (Table 3) with (Sigma-Aldrich Cat# A3562, RRID:AB_258091, 0.5ul/15 ml TBST).

OXT was observed at ~9 kDa (Fig. 1c), the core of un-glycosylated OXTR at ~36 kDa (Fig. 2e), and GAPDH at ~38 kDa. The molecular weights of OXT and GAPDH are in agreement with those predicted by the manufacturer. The molecular weight of un-glycosylated OXTR is within the range of 22–42 kDa predicted with ExPASy Server^64,65^ and NCBI^66^. Occasionally, OXTR bands were visible at ~48 and ~55 kDa, indicating glycosylation at one or two sites, which is common^45^.

After removing background density, the normalized expression was calculated by dividing the average intensity of the OXT or un-glycosylated OXTR band by that of the GAPDH band from the same horse that was run on the same gel.

### Immunohistochemistry on frozen samples

Samples were fixed and stained as described in Rapacz-Leonard^55^, except for the use of 0.5% BSA (AM2616, Thermo Fisher Scientific) for blocking non-specific binding. Briefly, Bloxall (SP-6000, Vector Laboratories) was used for blocking endogenous peroxidase, then 0.5% BSA and slides were incubated overnight with primary antibodies (Table 3: anti-OXT, anti-OXTR, anti-PTGS2). For detection, an ImmPRESS HRP Universal Antibody (Anti-Mouse IgG/Anti-Rabbit IgG, Peroxidase) Polymer Detection Kit (MP-7500, Vector Laboratories) for rabbit antibody and an ImmPRESS HRP Anti-Goat IgG (Peroxidase) Polymer Detection Kit (MP-7405, Vector Laboratories) for goat antibodies were used. Slides were photographed using a Zeiss microscope (Axio Imager.M2, Zeiss) with an AxioCam MRC 5 (Zeiss).

As positive controls, equine corpus luteum and the endometrium from a non-pregnant mare in late luteal phase were used. For negative controls, blocking peptides were used. Omission controls were performed with only antibody diluent (ab64211, Abcam). Replacement controls were performed with normal serum instead of primary antibody, using either rabbit serum (R9133, Sigma Aldrich) or goat serum (PK-4010, Vector Laboratories). No isotype control was performed because of the polyclonality of the antibodies.

### Enzyme immunoassays for PGE_2_ and PGF_2ɑ_

Samples were frozen in liquid nitrogen and stored at −80 °C. Extraction was performed as described in Siemieniuch^67^, who followed Tsang^68^. Briefly, the prostaglandins were extracted from samples with ethyl petroleum, and the supernatants were evaporated to dryness under nitrogen and reconstituted with 400 µL EIA buffer with 0.1% BSA. The extraction efficiencies were 74% for PGF_2ɑ_ and 72% for PGE_2_, as determined by performing the extraction with PGF_2α_ standard (ADI-931-069; ENZO Life Sciences Inc.) and PGE2 standard (ADI-931-001; ENZO Life Sciences Inc.), then measuring the concentration as below.

To measure the concentration of PGE_2_, a species-independent PGE_2_ EIA kit (ADI-931-001; ENZO Life Sciences Inc.) was used, which has intra- and inter-assay coefficients of variation of 6.7% and 12.2%, respectively. Because of the high concentration of PGE_2_, a 1:20 dilution of the samples in buffer solution was performed. To measure the concentration of PGF_2ɑ_, a species-independent PGF_2ɑ_ EIA kit (ADI-931-069; ENZO Life Sciences Inc.) was used, which has intra- and inter-assay coefficients of variation of 6.8% and 11.2%, respectively. With both kits, the manufacturer’s instructions were followed. For PGE_2_, the sensitivity was 8.26 pg/ml (range, 7.8–1,000 pg/ml); for PGF_2ɑ_, it was 0.98 pg/ml (range, 1.95–2,000 pg/ml). The content of PGE_2_ and PGF_2ɑ_ is reported as pg ml^−1^ g^−1^, adjusted for the dilution of PGE_2_.

### Statistical analyses

Full details of statistical methods are in Supplementary Note - Statistical analyses. Briefly, all data were log-transformed before further calculations and analyses^61,69^. For reporting, data was back-transformed to the original scale; thus, geometric means are reported.

Correlations between the amount of mRNA/protein/hormone and gestation days were assessed with Kendall’s tau (two-sided). Differences between mean expression of mRNA/protein/hormone in physiological parturition and FMR were tested with Welch’s t-test (two-tailed). Sensitivity analysis^70^ by bootstrapping the difference between medians^71^ indicated that potential deviations from normality did not affect the interpretation of Welch’s test (Supplementary Table S1).

To account for multiple comparisons, all 24 *p*-values were adjusted to maintain the false discovery rate below 5%^72^. After adjustment, results were considered significant at *p* < 0.05. Unadjusted 95% CIs for fold-changes are provided.

All statistical calculations were performed with R (v. 3.5.2^73^), employing the WRS package for R (v. 35^50^) for bootstrapping.

## Data Availability

The dataset analysed during the current study is available from Dryad Digital Repository (10.5061/dryad.fbg79cnr4)^74^.

## Supplementry information

Supplementry information.
