# Supplementary material for: The oxytocin-prostaglandins pathways in the horse (Equus caballus) placenta during pregnancy, physiological parturition, and parturition with fetal membrane retention
Source: Sci Rep. 2020 Feb 7;10:2089. doi: 10.1038/s41598-020-59085-1 (PMC7005845; doi:10.1038/s41598-020-59085-1)
Supplement: Supplementary file 1 — Supplementry information. [file 41598_2020_59085_MOESM1_ESM.pdf]

The oxytocin-prostaglandins pathways in the horse (*Equus caballus*) placenta during pregnancy, physiological parturition, and parturition with fetal membrane retention

Anna Rapacz-Leonard[1*], Mark Leonard[2], Małgorzata Chmielewska-Krzesińska[3],

Marta Siemieniuch[4], Tomasz E. Janowski[1]

[1] Department of Animal Reproduction with Clinic, Faculty of Veterinary Medicine, University of Warmia and Mazury in Olsztyn, Poland

[2] University of Warmia and Mazury in Olsztyn, Poland

[3] Department of Pathophysiology, Forensic Veterinary and Administration, Faculty of Veterinary Medicine, University of Warmia and Mazury in Olsztyn, Poland

[4] Research Station in Popielno / Department of Immunology and Pathology of Reproduction, Polish Academy of Science, Olsztyn, Poland

* Corresponding author

E-mail: anna.rapacz@uwm.edu.pl (ARL)

Supplementary Note - Statistical analyses

To avoid pseudoreplication, the mean amount of mRNA/protein/hormone in all samples from a horse was calculated before log-transformation. In order to maintain the statistical independence of groups, two mares that were sampled in physiological parturition in one year and in FMR in another were excluded from the FMR group, which had more horses (complete data deposited: Dryad Digital Repository (doi:10.5061/dryad.fbg79cnr4)[1].

Kendall's tau was chosen to quantify correlations because, unlike Pearson's *r*, it is robust to non-normality and can quantify non-linear associations[2]. Welch's t-test was chosen because it is more robust to heteroscedasticity than Student's t-test[2].

Because significance tests often cannot detect meaningful deviations from normality, sensitivity analysis of t-test results with a robust method is recommended[3]. Thus, the difference between medians was bootstrapped (2000 replicates), which is a recommended technique for sample sizes like ours[4]. Differences between medians were within the 95% confidence intervals of differences between means, and conclusions about statistical significance were the same with both methods (Supplementary Table S1); therefore, the results of Welch's test were reported.

Supplementary Table S1. Test statistics, *P*-values, 95% confidence limits, and sensitivity analysis.

substance	name	tissue	df	t	FC (means)	LCL (means)	UCL (means)	FC (medians)	*p*-value (means)	*p*-value (medians)
mRNA	*OXTR*	A	18.71	-1.08	1.5	-1.4	3.2	1.4	0.295	0.480
		E	9.20	-0.84	3.5	-8.3	102.1	-1.1	0.422	0.974
	PTGS2	A	15.18	4.68	-14.7	-49.8	-4.3	-18.5	0.0003	<0.0005
		E	10.84	1.85	-8.4	-105.7	1.5	-68.7	0.092	0.267
peptide	OXT	A	5.74	-1.65	3.7	-1.9	27.0	1.4	0.152	0.188
		E	7.35	-0.10	1.2	-30.1	41.1	-1.4	0.922	0.930
protein	OXTR	A	6.31	-1.74	1.8	-1.2	3.9	1.0	0.130	0.677
		E	8.29	-1.52	1.7	-1.3	4.0	2.4	0.165	0.188
hormone	PGE_2	A	14.45	-1.17	1.4	-1.3	2.4	1.1	0.260	0.732
		E	8.96	-0.35	1.2	-3.3	5.1	-1.4	0.732	0.579
	$PGF_{2\alpha}$	A	13.79	-1.21	1.5	-1.4	3.0	-1.3	0.246	0.869
		E	8.36	0.21	-1.2	-7.3	5.2	-1.4	0.835	0.796

Sensitivity of Welch's t-test results to outliers or non-normality of data was assessed by bootstrapping the difference between medians. Fold-changes between medians were within 95% confidence intervals for fold-changes between means, and conclusions about statistical significance were the same with both methods. Unadjusted *p*-values are presented here.

Abbreviations: OXTR, oxytocin receptor; PTGS2, prostaglandin endoperoxide synthase-2; OXT, oxytocin; PGE_2, prostaglandin E2; $PGF_{2\alpha}$, prostaglandin F2 alpha; A, allantochorion; E, endometrium; df, degrees of freedom; t, test statistic; FC, fold-change; LCL, lower 95% confidence limit; UCL, upper 95% confidence limit

Supplementary Table S2. Prostaglandin E2 and prostaglandin F2 alpha content.

hormone	tissue	group	n	geometric mean ($pg\ ml^{-1}\ g^{-1}$)	range ($pg\ ml^{-1}\ g^{-1}$)
PGE_2	allantochorion	PREG	5	207	94–377
		PHYS	9	278	132–612
		FMR	8	376	230–739
	endometrium	PREG	5	24	13–47
		PHYS	6	61	9–185
		FMR	5	76	24–353
$PGF_{2\alpha}$	allantochorion	PREG	5	20	9–32
		PHYS	9	174	27–445
		FMR	8	260	140–645
	endometrium	PREG	5	16	6–39
		PHYS	6	38	5–481
		FMR	5	32	7–89

Abbreviations: PGE_2, prostaglandin E_2; $PGF_{2\alpha}$, prostaglandin $F_{2\alpha}$; PREG, pregnancy (days 90–240); PHYS, physiological parturition; FMR, parturition with fetal membrane retention; n, number of mares

Supplementary Table S3. Sequences of PCR products.

**Gene symbol and sequence accession number**	**Sequence accession number**	**Percent identity with NCBI sequence**	**PCR sequence**
Glyceraldehyde-3-phosphate dehydrogenase, reference gene<br>*GAPDH*	NM_001163856.1	100%	GTCAAGCTCATTTCCTGGTATGACAATGAATTTGGCTACAGCAATAGGGTGGT<br>GGACCTTATGGCCCACATGGCCTCCAAGGAGTAAGAGCCCCCTGGACCACCAA<br>TCACCCAGCAA
Oxytocin receptor<br>*OXTR*	XM_023620040.1<br>variant X1<br>XM_023620041.1<br>variant X2<br>XM_014731360.2<br>variant X3	98%	TTCATCATCGTGCTGGCCTTCATCGTGTGCTGGACGCCATTCTCTCGTGCAGAT<br>GTGGAGCGTCTGGGACCCCAACGCGCCCAAGGAAGCCTCGGCTTTCA
Prostaglandin-endoperoxide synthase 2<br>*PTGS2*	NM_001081775.2	100%	TATGGTGAAACTTTGGATAGACAGCATAAACTGCGCCTTTTCAAGGACGGAAA<br>AATGAAATATCAGATCATTAATGGCGAGGTGTATCCGC

Sequences from bands excised after electrophoresis after conventional PCR.

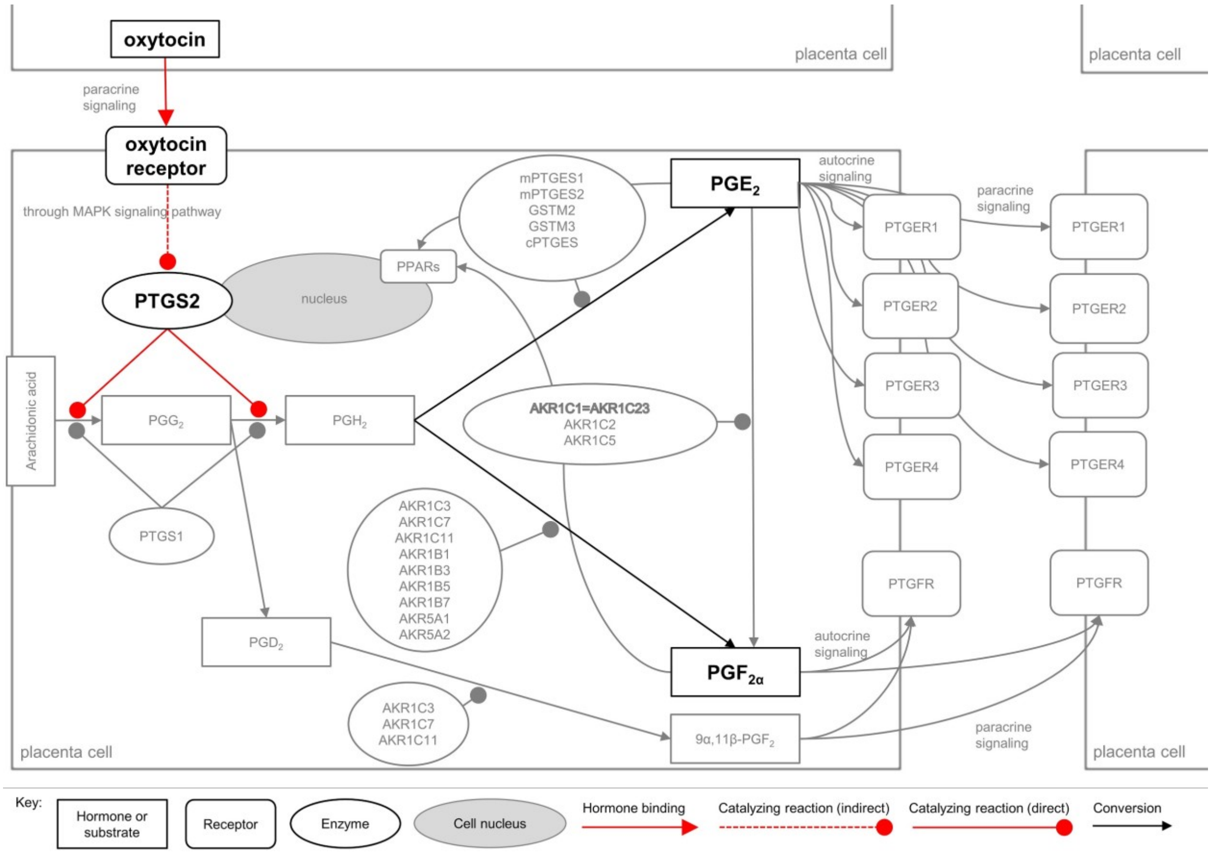

Supplementary Fig. S1. Simplified diagram of pathways leading from oxytocin release to prostaglandin signaling in placental cells. This diagram was based on[5–14].

Abbreviations: PTGS2, prostaglandin endoperoxide synthase-2; PTGS1, prostaglandin endoperoxide synthase-1; PGG_2, prostaglandin G_2; PGH_2, prostaglandin H_2; PGD_2, prostaglandin D_2; AKR1C1, aldo-keto reductase family 1 member C1; AKR1C23, aldo-keto reductase family 1 member C23; AKR1C2, aldo-keto reductase family 1 member C2; AKR1C3, aldo-keto reductase family 1 member C3; AKR1C5, aldo-keto reductase family 1 member C5; AKR1C7, aldo-keto reductase family 1 member C7; AKR1C11, aldo-keto reductase family 1 member C11; AKR1B1, aldo-keto reductase family 1 member B1; AKR1B3, aldo-keto reductase family 1 member B3; AKR1B5, aldo-keto reductase family 1 member B5; AKR1B7, aldo-keto reductase family 1 member B7; AKR5A1, aldo-keto reductase family 5 member A1; AKR5A2, aldo-keto reductase family 5 member A2; 9α,11β-PGF_2, 9α,11β-prostaglandin F_2; mPTGES1, microsomal prostaglandin E2 synthase 1; mPTGES2, microsomal prostaglandin E2 synthase 2; GSTM2, glutathione S-transferase Mu 2; GSTM3, glutathione S-transferase M3; cPTGES, cytosolic prostaglandin E2 synthase; PGE_2, prostaglandins E_2; PTGER1, prostaglandin E2 receptor EP1; PTGER2, prostaglandin E2 receptor EP2; PTGER3, prostaglandin E2 receptor EP3; PTGER4, prostaglandin E2

receptor EP4; $PGF_{2\alpha}$, prostaglandin $F_{2\alpha}$, PTGFR, prostaglandin F2α receptor, PPARs, peroxisome proliferator-activated receptors.

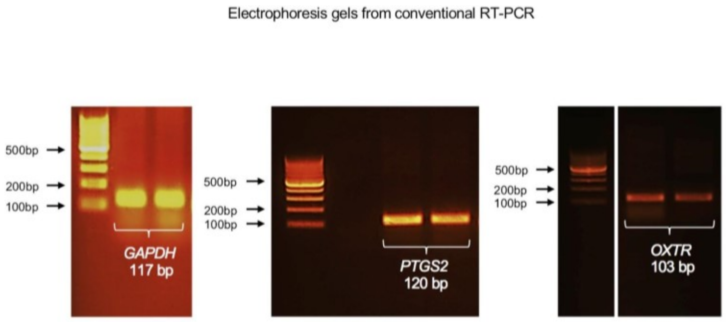

Supplementary Fig. S2. Electrophoresis gels from conventional RT-PCR. The figure shows electrophoresis gels from conventional RT-PCR that was run with the same primers later used in quantitative RT-PCR. Conventional PCR was run as a step in preparation of products for sequencing. Each lane shows one strong band at the predicted length (117bp for *GAPDH*, 120bp for *PTGS2* and 103bp for *OXTR*), which confirms the specificity of the primers. (White line in the gel with OXTR indicates that this gel was cut and bands not relevant to this study were removed.)

Abbreviations: *GAPDH*, glyceraldehyde-3-phosphate dehydrogenase; *OXTR*, oxytocin receptor; *PTGS2*, prostaglandin-endoperoxide synthase 2; bp, base pair.

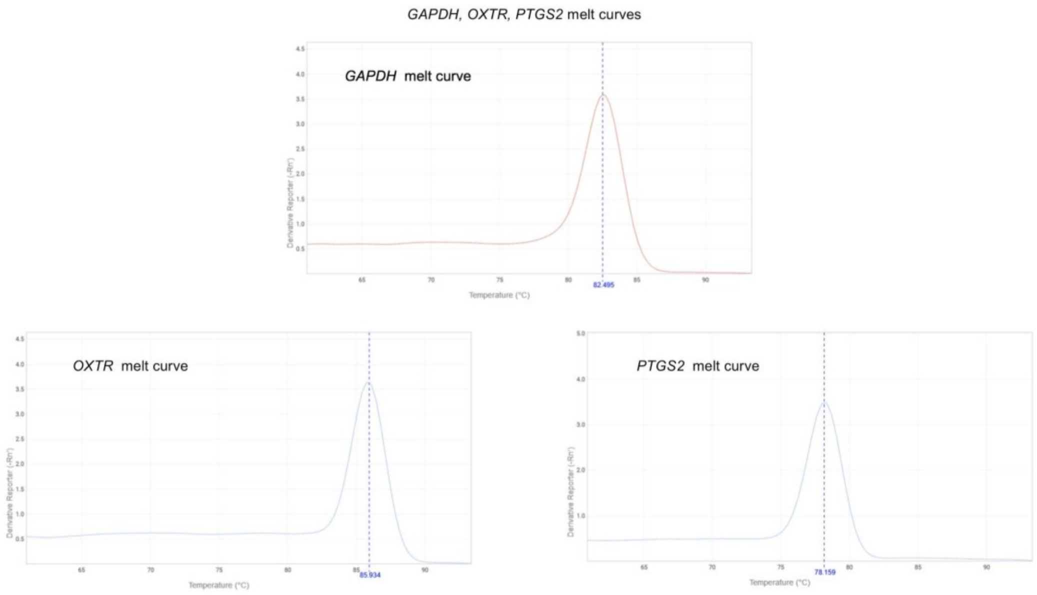

Supplementary Fig S3. *GAPDH, OXTR, PTGS-2* melt curves from RT-qPCR. The figure shows melt curves of primers for quantitative RT-PCR. Note that each well has only one melt curve, indicating the specificity of the primers.

Abbreviations: *GAPDH*, glyceraldehyde-3-phosphate dehydrogenase; *OXTR*, oxytocin receptor; *PTGS2*, prostaglandin-endoperoxide synthase 2.

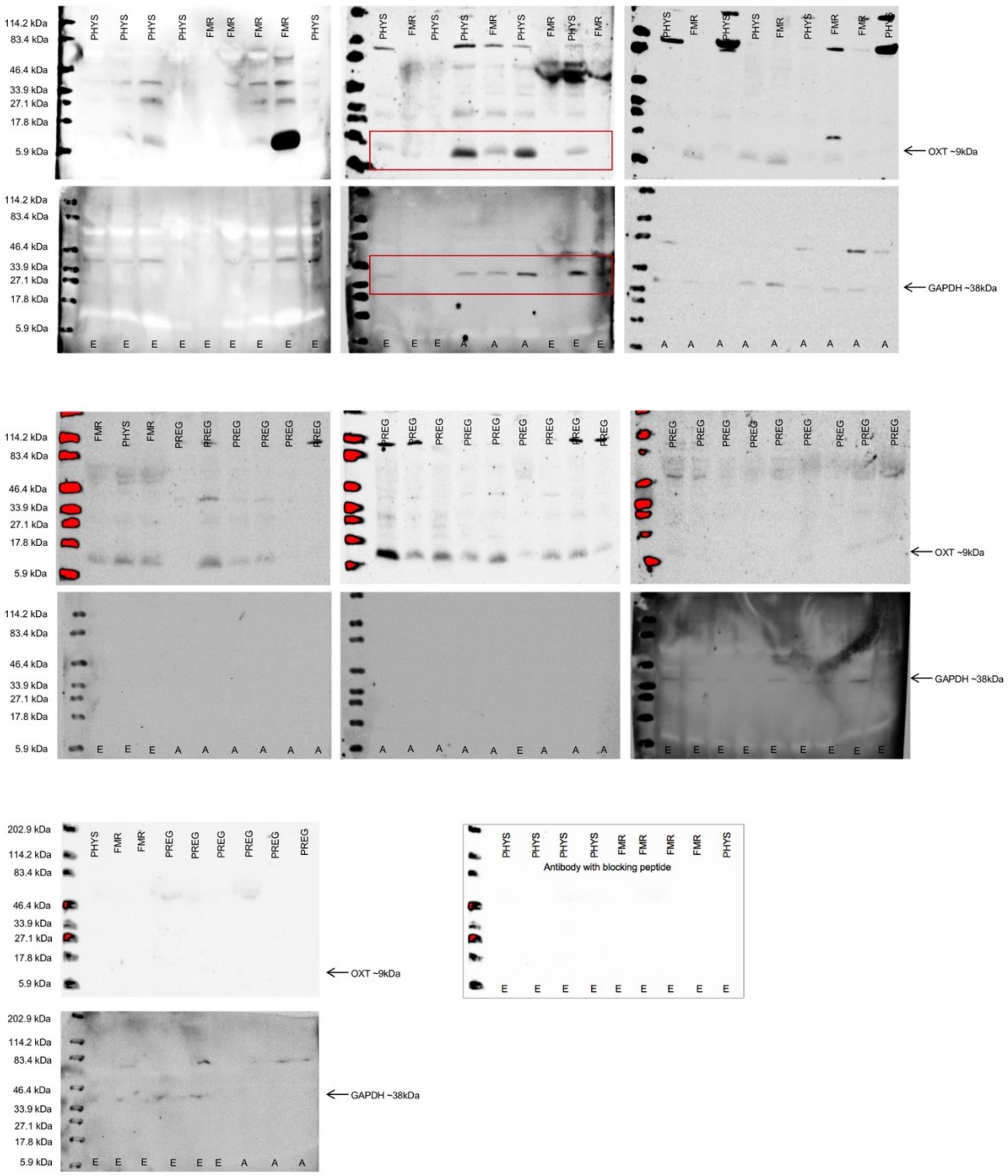

Supplementary Fig. S4. Uncropped western blots for oxytocin.

Red boxes indicate the blot that was cropped and presented in figure 1c.

Abbreviations: GAPDH, glyceraldehyde-3-phosphate dehydrogenase; OXT, oxytocin; kDa, kilodalton; PHYS, physiological parturition; FMR, parturition with fetal membrane retention; A, allantochorion; E, endometrium.

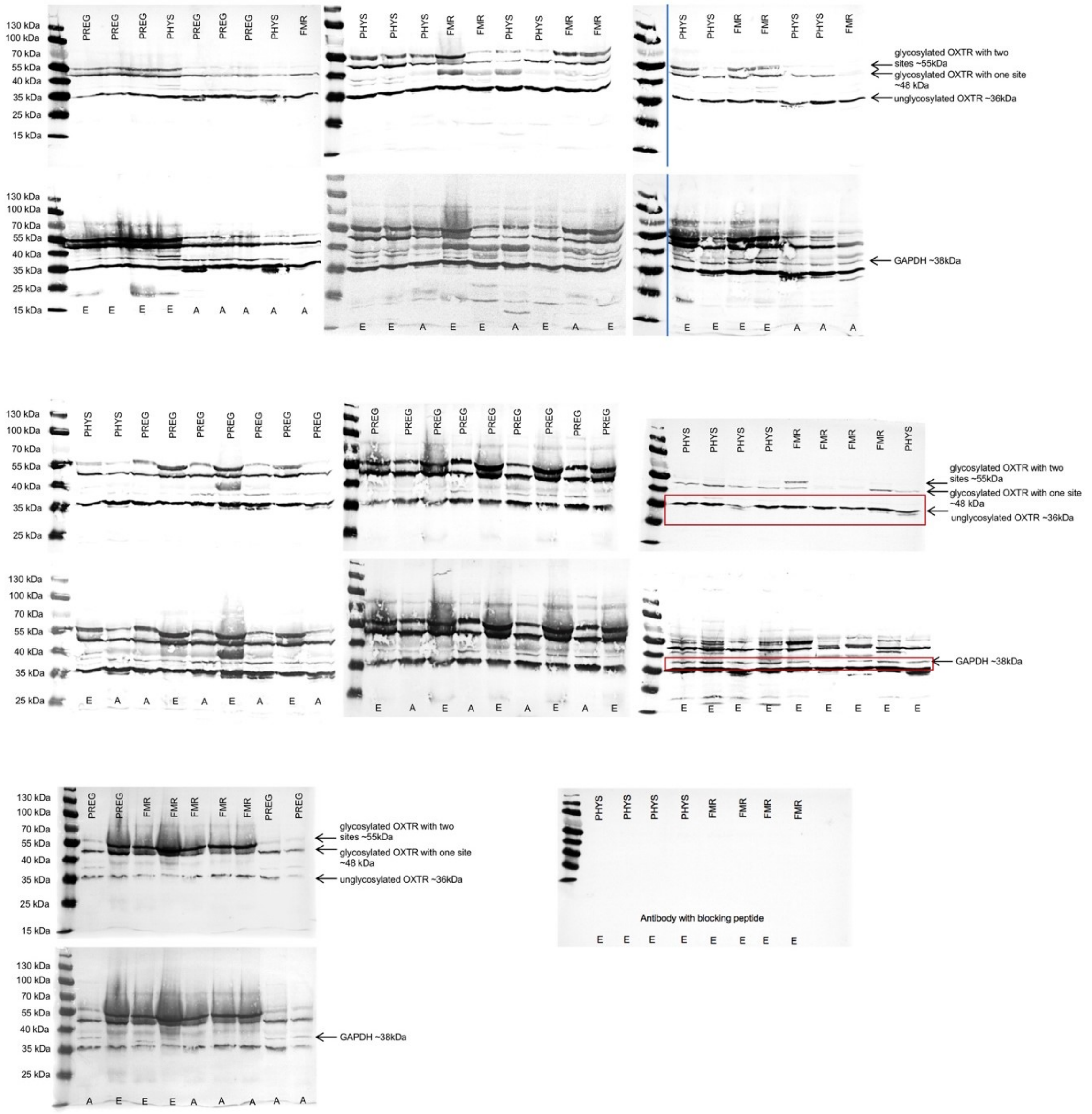

Supplementary Fig. S5. Uncropped western blots for oxytocin receptor.

Red boxes indicate the blot that was cropped and presented in figure 2e. Blue vertical lines in the two top blots on the right indicate that these blots were cut and lanes not relevant to this study were removed. Please note that in this method (colorimetric western blotting) there is no stripping step before incubation with GAPDH antibody. Therefore, on blots with GAPDH bands, OXTR bands are also visible.

Abbreviations: GAPDH, glyceraldehyde-3-phosphate dehydrogenase; OXTR, oxytocin receptor; kDa, kilodalton; PHYS, physiological parturition; FMR, parturition with fetal membrane retention; A, allantochorion; E, endometrium.

References:

1. Rapacz-Leonard, A., Leonard, M., Chmielewska-Krzesińska, M., Siemieniuch, M. & Janowski, T. The oxytocin-prostaglandins pathways in the horse (Equus caballus) placenta during pregnancy, physiological parturition, and parturition with fetal membrane retention. *Dryad Dataset* (2019). doi:10.5061/dryad.fbg79cnr4
2. Wilcox, R. R. *Understanding and applying basic statistical methods using R*. (John Wiley & Sons, 2017).
3. Field, A. P. & Wilcox, R. R. Robust statistical methods: A primer for clinical psychology and experimental psychopathology researchers. *Behav. Res. Ther.* **98**, 19–38 (2017).
4. Wilcox, R. R. & Rousselet, G. A. A Guide to Robust Statistical Methods in Neuroscience. in *Current Protocols in Neuroscience* **82**, 8.42.1-8.42.30 (John Wiley & Sons, Inc., 2018).
5. Gibb, W. The role of prostaglandins in human parturition. *Ann. Med.* **30**, 235–241 (1998).
6. Mancini, J. A. *et al.* Cloning, Expression, and Up-regulation of Inducible Rat Prostaglandin E Synthase during Lipopolysaccharide-induced Pyresis and Adjuvant-induced Arthritis. *J. Biol. Chem.* **276**, 4469–4475 (2001).
7. Slater, D. M., Zervou, S. & Thornton, S. Prostaglandins and Prostanoid Receptors in Human Pregnancy and Parturition. *J Soc Gynecol Investig* **9**, 118–124 (2002).
8. Trebino, C. E. *et al.* Impaired inflammatory and pain responses in mice lacking an inducible prostaglandin E synthase. *Proc. Natl. Acad. Sci.* **100**, 9044–9049 (2003).
9. Sandig, H., Andrew, D., Barnes, A. A., Sabroe, I. & Pease, J. 9α,11β-PGF2 and its stereoisomer PGF 2α are novel agonists of the chemoattractant receptor, CRTH2. *FEBS Lett.* **580**, 373–379 (2006).
10. Dozier, B. L., Watanabe, K. & Duffy, D. M. Two pathways for prostaglandin F2α synthesis by the primate periovulatory follicle. *Reproduction* **136**, 53–63 (2008).
11. Kabututu, Z. *et al.* Prostaglandin F2 Synthase Activities of Aldo-Keto Reductase 1B1, 1B3 and 1B7. *J. Biochem.* **145**, 161–168 (2009).
12. Nagata, N., Kusakari, Y., Fukunishi, Y., Inoue, T. & Urade, Y. Catalytic mechanism of the primary human prostaglandin F2α synthase, aldo-keto reductase 1B1 - Prostaglandin D2 synthase activity in the absence of NADP(H). *FEBS J.* **278**, 1288–1298 (2011).
13. Pépin, N. L., Chapdelaine, P., Rodriguez, Y., Tremblay, J. P. & Fortier, M. A.

Generation of human endometrial knockout cell lines with the CRISPR-Cas9 system confirms the prostaglandin F2α synthase activity of aldo-ketoreductase 1B1. *Mol. Hum. Reprod.* **20**, 650–663 (2014).

14. Chatterjee, O. *et al.* An overview of the oxytocin-oxytocin receptor signaling network. *J. Cell Commun. Signal.* **10**, 355–360 (2016).
